# Ecological status improvement over a decade along the Ligurian coast according to a macroalgae based index (CARLIT)

**DOI:** 10.1371/journal.pone.0206826

**Published:** 2018-12-31

**Authors:** Gina De La Fuente, Mariachiara Chiantore, Federico Gaino, Valentina Asnaghi

**Affiliations:** 1 Dipartimento di Scienze della Terra dell'Ambiente e della Vita–DISTAV, Università degli Studi di Genova, Genova, Italy; 2 Consorzio Nazionale Interuniversitario per le Scienze del Mare–CoNISMa, Rome, Italy; 3 Agenzia Regionale per la Protezione dell’Ambiente Ligure–ARPAL, Genova, Italy; University of Waikato, NEW ZEALAND

## Abstract

According to the Water Framework Directive, within 2015 European Union countries must reach and maintain the “good” Ecological Status (ES), quantified through indices based on key biological elements as indicators. Along the Ligurian shallow rocky coasts (NW Italy), a macroalgae based index (CARtography of LITtoral and upper-sublittoral benthic communities, CARLIT), calibrated according to national characteristics and management needs, has been applied by the Regional Environmental Agency over the last ten years. In 2015, at least a “good” ES was achieved in all Ligurian water bodies except one, located in the Eastern Ligurian coastline, characterized by the lack of the most sensitive species, *Cystoseira amentacea var*. *stricta*. A general ES improvement has been observed along the Ligurian coastline, also in comparison with other quality indices (macroinvertebrates and fecal bacteria), and in particular in the Genoa water body, as proved by a relevant increase of *C*. *amentacea* abundance, probably as a consequence of enhancement in wastewater treatments. In the present study, the reliability of the observed improvement of the ES over a decade has been assessed, teasing apart intra-seasonal and operator-related variability. These results support the reliability of monitoring procedures carried out though the CARLIT Index and highlight the need and the effectiveness of reduction measures for anthropogenic impacts in order to achieve the ES required by European directives.

## Introduction

The Water Framework Directive (WFD, 2000/60/EC) adopted by the European Community in 2000 with the goal of maintaining and improving the quality of aquatic environments, in its first management cycle, requires that member states achieve and maintain a “good” Ecological Status (ES) of all water bodies by 2015, postponed to 2021 and 2027 for the second and third management cycles. The ES has to be quantified applying indices based on appropriate key biological elements as indicators. A ratio between observed values and reference ones (EQR, Ecological Quality Ratio) of the indicator allows to rank water bodies from 0 (bad ecological quality) to 1 (high ecological quality), identifying different ES classes: bad, poor, moderate, good and high.

The CARLIT Index, CARtography of LITtoral and upper-sublittoral benthic communities [1], assesses coastal water quality in the Mediterranean Sea using intertidal macroalgal assemblages as key biological elements: the EQR is calculated dividing the Ecological Quality Value, scored between 1 and 20 attributed according to the dominant macroalgal community, by the reference value adopted for the given geomorphological relevant situation [1]. The selection of a proper reference site is fundamental for the CARLIT Index calculation [2]. This index is widely used in the EU Mediterranean countries: Spain (Catalan coast [1], Alboran Sea [3]); France (Southern coast and Corsica [4]); Italy ([5], Ligurian Sea [2, 6], Gulf of Naples [7], Northwestern Adriatic Sea [8], Sardinia [9], Tyrrhenian islands [10]); Croatia (Northeastern Adriatic Sea [11], Malta [12]) and in three non-EU countries: Albania [13], Tunisia [14] and Lebanon [15].

The rationale of CARLIT Index is the different sensitivity to environmental stresses displayed by macroalgal assemblages along rocky shores [16–24]. The lowest levels of sensitivity correspond to the opportunistic species (mainly Ulvales order [25,26]), the intermediate values correspond to the stress-tolerant species (mainly Corallinales order [27,28]) and the highest sensitivity levels refer to the species belonging to the *Cystoseira* genus (Fucales order) [27,29,30]. *Cystoseira* species display a large size and complex structure with an arborescent thallus: where well-developed, these species can form forests, which play an important role as ecosystem engineers, supporting a highly structured and diversified macroalgal assemblage and providing shelter (refuge) and food for marine invertebrates and for juvenile fish [31,32].

At present, the CARLIT Index is regularly implemented in Italy, where it has been calibrated according to national characteristics, as reported in the ISPRA (Italian National Institute for Environment Protection and Research) methodological report ([28] http://www.isprambiente.gov.it/it/archivio/notizie-e-novita-normative/notizie-ispra/anno-2009/pubblicazione-amb-mar). The first implementation of CARLIT Index was performed in Liguria, in 2005–2007 [2,6]: in the following years, CARLIT monitoring has been carried out by the Regional Environmental Agency (ARPAL) as part of the institutional monitoring programme (Environmental and Sea Defense Ministry–Monitoring Programme, ex L. 979/82). According to ISPRA methodological report, the CARLIT Index should be calculated uninterruptedly along the entire rocky coast. As an alternative, in regional studies, a hierarchical design, encompassing large and representative stretches of coast for each water body, can also be applied [6]. In the Ligurian Sea, for example, the CARLIT Index is calculated by ARPAL following a hierarchical design.

The use of the CARLIT Index as a monitoring tool for the Regional Agencies has several advantages, because of non-destructive sampling, reduced costs and easy-to-acquire taxonomic expertise [1]. Yet, the methodology has the disadvantage that needs to be implemented in a specific and limited time-frame, due to the strongly seasonal development of macroalgal assemblages. In fact, the CARLIT monitoring must be performed in late spring (April—June), when macroalgae reach their growth peak. Since the application of the CARLIT Index along the entire regional coast may be quite long due to unstable sea conditions in spring time, the seasonality of macroalgal growth may be considered a potential bias in the comparison of sites sampled at different times within the spring season. Particularly, *Cystoseira* spp. (the most sensitive species), show strong variability in their thalli length, that can potentially confound the attribution of the correct community category, affecting the CARLIT Index calculation. Additionally, a long term implementation of the index necessarily implies collection of data by different operators, that could represent an additional source of uncertainty, particularly in the attribution of intermediate categories.

The present research focuses on the assessment of the Ecological Status along the Ligurian coastline over a decade through the CARLIT Index, teasing apart the effects of intra-seasonal variability within the period of implementation (spring) and sampling operator effect. The objective of this approach is to verify if the correct attribution of the ES class may be affected by such potential sources of variability.

## Material and methods

### Study site

The Ligurian region is located in the NW of Italy (43°47’01”N-7°32’03”E, 44°2’38”N-10°1’1”E) and encompasses more than 300 km of coast. The main superficial current along the Ligurian coast is formed by the convergence of the Tyrrhenian current and the West-Coast Corsica current, moving along the shore from East to West. The climate of the Ligurian region is strongly influenced by the proximity of the mountains that protect it from the cold northern winds and the presence of the sea that mitigates the temperatures even if it makes the region very humid. Rainfalls along the eastern coast can reach 1300 mm of rain per year, while the western coast is less rainy (700 mm of rain per year). Predominant winds are of western and southern origin. Along the coasts, the marine waters damp the seasonal and daily thermal excursions: in the summer temperatures occasionally exceed 30°C and only in the colder winters temperature fall below 10°C.

Three of the largest Italian harbors are located in the region: Savona Vado (SV), Genova (GE) and La Spezia (SP), which carry out commercial, industrial and recreational activities. These harbors are located in the three Ligurian largest urbanized areas, particularly Genoa, with approximately 700,000 inhabitants.

The Ligurian coast represents the ideal site for this validation study, because it has been the pilot area for the definition of the Italian CARLIT methodology and, being the first place where this index was applied, provides the longest temporal dataset.

### Sampling strategies

In the present study, two sampling approaches were implemented in order to assess i) the ecological status of Ligurian region water bodies over a decade, considering operator-associated variability and ii) the potential intra-seasonal variability of the Index.

#### Ecological status over a decade

Since 2006, 16 water bodies (WB) are sampled for ecological status assessment along the Ligurian rocky coast applying the macroalgae based index, CARLIT. The area of each water body extends up to 3 km from the coast and in any case encompasses the 50 m bathymetric. The WBs were identified by ARPAL and Liguria Region mainly considering the following factors (available as GIS information in the Regional Cartographic System): morphology of the coast; presence and type of marine angiosperms; main pressures insisting on the coast, both punctual (discharges, pipelines, ports) and widespread (land use); presence of marine protected areas; bathing areas; fish-farming areas.

According to ISPRA methodological report [28], the CARLIT index is calculated by ARPAL in these WBs. Each WB is divided into three areas: West, Central and East, each area composed by 20–30 sectors (50 meters each), depending on the length of the rocky shore in each WB.

Each year, a subset of the water bodies is monitored so that each WB is visited once every three years.

**Fig 1:**
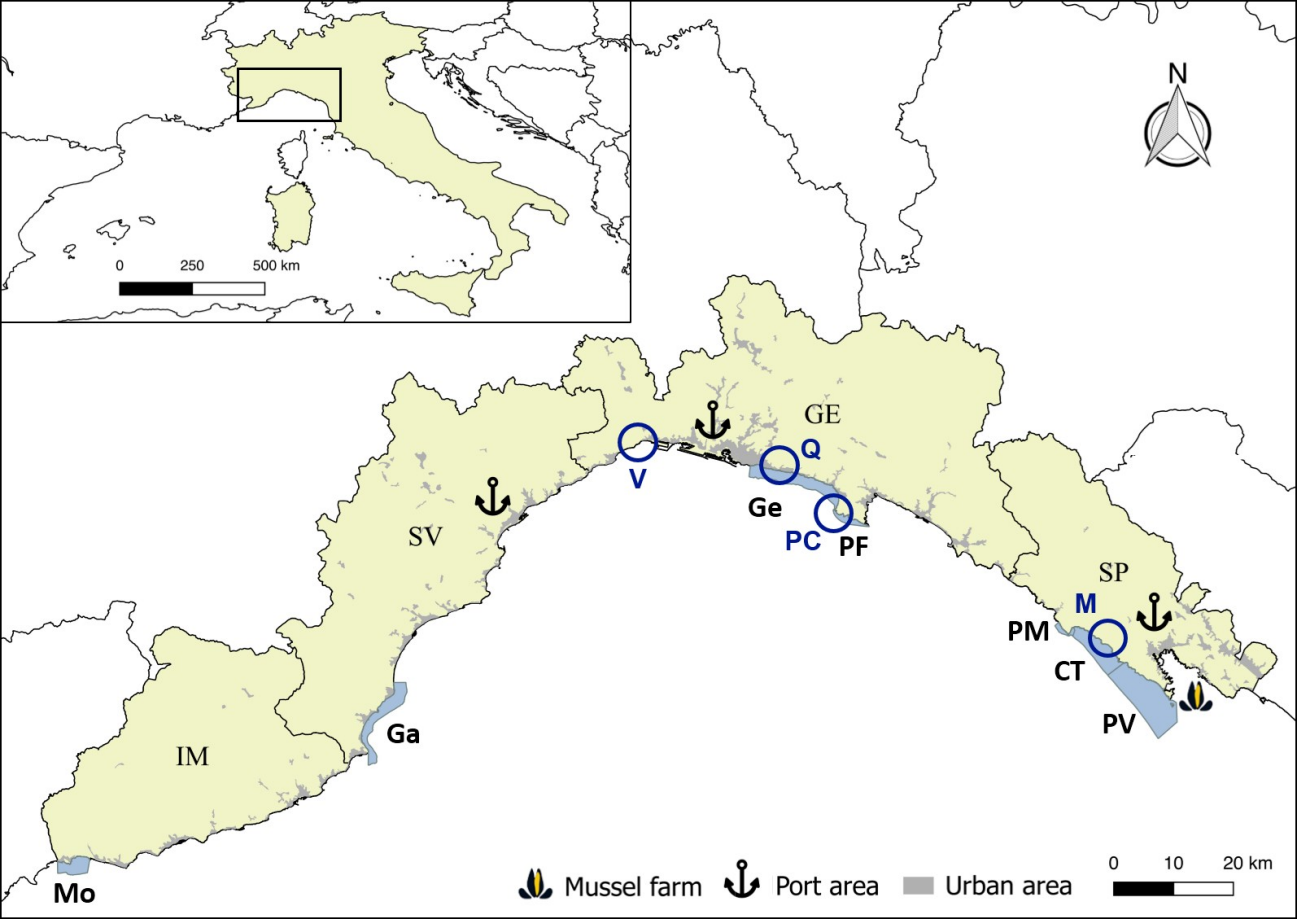
Geographical distribution of the different sites along the Ligurian coast (NW Med). The CARLIT Index was calculated in the WBs highlighted in pale blue along the coast: 1- Capo Mortola (Mo); 2- Laigueglia-Albenga (Ga); 3- Genova-Camogli (Ge); 4- Portofino (PF); 5- Punta Mesco (PM); 6- Cinque Terre (CT); 7- Portovenere (PV); the intra-seasonal variability study has been performed in Sites marked with circles: V- Vesima; Q- Quarto dei Mille; PC- Punta Chiappa; M- Manarola. IM (Imperia), SV (Savona), GE (Genova) and SP (La Spezia) are the four Ligurian provinces. Main harbors and La Spezia mussel mariculture are reported with symbols.

In the present study, we took into account the ecological status assessed through the CARLIT methodology [1] during spring seasons, within the timeframe from 2006 to 2015, in seven WBs (Fig 1): Capo Mortola (Mo; 43°47’01”N-7°32’03”E, 43°47’22”N-7°35’50”E), Laigueglia-Albenga (Ga; 43°57’29”N-8°10’26”E, 44°3’38”N-8°13’30”E), Genova-Camogli (Ge; 44°23’24”N-8°58’12”E, 44°19’21”N-9°8’42”E), Portofino (PF; 44°19’21”N-9°8’42”E, 44°17’55”N-9°13’8”E), Punta Mesco (PM; 44°9’6”N-9°36’53”E, 44°8’37”N-9°38’47”E), Cinque Terre (CT; 44°8’37”N-9°38’47”E, 44°5’25”N-9°45’1”E) and Portovenere (PV; 44°5’25”N-9°45’1”E, 44°2’0”N-9°50’48”E). These WBs have been selected in order to be representative of the entire Ligurian coastline: two are located in the western (Mo, Ga), two in the central (Ge, PF) and three in the eastern side (PM, CT, PV). Additionally, sites are differently affected by anthropogenic pressures, since they are located in harbor areas (Ge, PV), close to mussels farm (PV) or within areas under different levels of protection (Mo, Ga, PF, PM, CT). All sampling sites are located on public land: for Portofino and Cinque Terre MPAs (PF, PM, CT), a specific permission has been provided by the competent authorities (Management Consortium of the Portofino Marine Protected Area and Cinque Terre National Park, respectively), in the framework of ARPAL institutional monitoring programme. All the other sites are not subject to particular protection restrictions. The locations are not privately-owned or protected in any way. The sampling strategy applied is not-destructive, meaning that no species has been collected or damaged.

The rocky coast was covered with a small boat and the dominant macroalgal community and geomorphological features of each sector were recorded on a cartographic support using the Quantum Geographical Information System (QGIS). Each water body was sampled in one day; different teams of experts, applying the identical methodology, were involved along the considered time-frame. The sampling procedure is non-destructive and does not involve the collection of endangered or protected species, as well as of any other species.

In addition, two pressure indices, the Land Uses Simplified Index (LUSI [33]) and the modified LUSI Index (MA-LUSI-WB [34]), were calculated for each WB in order to relate anthropogenic pressures and the EQR values.

#### Intra-seasonal variability

*Cystoseira* species, one of the most sensitive community category in the CARLIT Index calculation (Table 1), may be variable in terms of morphological features and percent cover along the time-frame considered for CARLIT index implementation (the whole spring season). An intra-seasonal study was carried out in order to verify if this variability can affect the attribution of the correct community category and, consequently, of the Ecological Status (ES).

**Table 1:** Summarized description and sensitivity levels (20 to 1, decreasing from high to low) of the main community categories as reported in the methodological contribution published by ISPRA (modified from [28]).

Community category	Description	SL	Comm. Acronim
Trottoir^a^	Large organogenic build-ups of *Lithophyllum byssoides*, *Lithophyllum trochanter*, *Dendropoma*^*b*^	**20**	TR
*Cystoseira brachycarpa* / *crinita* / *elegans*	Community dominated by *Cystoseira brachycarpa* / *crinita* / *elegans*	**20**	CB
*Cystoseira* sheltered	Community dominated by *Cystoseira foeniculacea* / *barbata* / *humilis* / *spinosa*	**20**	Cs
*Cystoseira amentacea* / *mediterranea* 5	Continuous belt of *Cystoseira amentacea* / *mediterranea*	**20**	CA5
*Cystoseira amentacea* / *mediterranea* 4	Almost continuous belt of *Cystoseira amentacea* / *mediterranea*	**19**	CA4
*Cystoseira amentacea* / *mediterranea* 3	Abundant patches of dense stands of *Cystoseira amentacea* / *mediterranea*	**15**	CA3
*Cystoseira amentacea* / *mediterranea* 2	Abundant scattered plants of *Cystoseira amentacea* / *mediterranea*	**12**	CA2
*Cystoseira compressa*	Community dominated by *Cystoseira compressa*	**12**	CC
*Cystoseira amentacea* / *mediterranea* 1	Rare scattered plants of *Cystoseira amentacea* / *mediterranea*	**10**	CA1
Dictyotales / Stypocaulaceae	Community dominated by *Padina* / *Dictyota* / *Dictyopteris* / *Taonia* / *Stypocaulon*	**10**	DS
*Corallina*	Community dominated by *Corallina* spp. (including *Ellisolandia elongata*)	**8**	Cor
Encrusting corallinales	Community dominated by *Lithophyllum incrustans*, *Neogoniolithon brassica-florida* and other encrusting corallines	**6**	EC
Mussels	Community dominated by *Mytilus galloprovincialis*	**6**	Mgal
*Pterocladiella / Ulva / Schizymenia*	Community dominated by *Pterocladiella / Ulva / Schizymenia*	**6**	Ulva
Green algae	Community dominated by *Ulva* and / or *Cladophora*	**3**	GA
Blue greens	Community dominated by *Cyanobacteria* and *Derbesia tenuissima*	**1**	BG
*Posidonia* reef	Barrier and fringing reefs of *Posidonia oceanica*	**20**	Pos
*Cymodocea nodosa*	Superficial *Cymodocea nodosa* meadows	**20**	Cym
*Zostera noltii*	Superficial *Zostera noltii* meadows	**20**	Zos

^a^Except for the category Trottoir, which is generally found in the mediolittoral zone, all the other categories only have be taken into account when present in the infralittoral fringe zone.

^b^*Dendropoma* forms organogenic build-ups typical of Sicily and other South Italian regions.

In the case of rare scattered plants of *Cystoseira amentacea* / *mediterranea*, the dominant community also has to be noted down. (Sensitivity level—SL: average value).

In case of sectors equally dominated by two different community categories, the average value between the two is taken into account (e.g. Cor+Mgal: SL = 7)

Sampling was performed during spring 2015 at four out of the seven WBs considered for the long-term comparison (Fig 1): Vesima (V; 44°25'25"N-8°44'16"E), Quarto dei Mille (Q; 44°23'22"N-8°59'31"E), Punta Chiappa (PC; 44°19'21"N-9°08'46"E) and Manarola (M; 44°06'26"N-9°43'33"E). These sites have been selected as representative of different ES levels, different protection regimes (two are within MPAs) or accessibility to humans (proximity to urban areas).

The four sites were chosen for different reasons: 1- they display different ES scores, 2- are spread along the coast in order to encompass, as much as possible, the extension (and associated natural variability) of the Ligurian rocky coast, 3- are different in terms of urbanization impact (Vesima and Quarto dei Mille are differently affected by the Genoa urban center; Punta Chiappa and Manarola are located inside Marine Protected Areas).

Sampling was performed monthly from March to June, in order to encompass the period of maximum macroalgal development. At each site, three 50 meters long sectors (a stretch of coast of 150 m) were sampled according to the CARLIT methodology, identifying the appropriate community category in each one (Table 1, according to [28]). According to Ballesteros et al. (2007), the EQR for each site was obtained as the ratio between the value corresponding to the community category (Table 1) characteristic of the site and the reference value (EQi) for its geomorphological relevant situation (Table 2).

**Table 2:** Ecological quality values (EQi) for the six geomorphological relevant situations (GRS) in reference conditions.

GRS(i)^a^	Coastal morphology	Type of substrate	EQi
**1**	Decimetric blocks	Natural	12.2
**2**	Low coast	Natural	16.6
**3**	High coast	Natural	15.3
**4**	Decimetric blocks	Artificial	12.1
**5**	Low coast	Artificial	11.9
**6**	High coast	Artificial	8.0

^a^GRS are described depending on their coastal morphology and type of substrate.

### Data analysis

#### CARLIT Index calculation

The CARLIT Index was calculated obtaining the ecological quality ratio (EQRs) values using the CARLIT package [35] with the free software R (R Development Core Team 2014, Version 3.1.0), which provides averaged values for each water body. The CARLIT package requires three datasets. The first one (containing actual data collected in the field) includes data columns in the following order: Site, Morphology (Decimetric blocks, Low coast, High coast), NatArt (natural or artificial substrate), Length (sector length) and Community (Categories of macroalgal communities). The second one is the Sensitive Level (SL) dataset, reporting the sensitivity of each macroalgal community category, as reported in the methodological contribution published by ISPRA (see Table 1). The third one is the reference dataset, reporting reference values for each geomorphological relevant situation (according to [1]). The benefit provided by the CARLIT package in R is that the two latter datasets may be easily adapted to the characteristics of each region/basin, according to the occurrence and the abundance of the commonest upper-infralittoral macroalgal communities or to regional/national management requirements.

The EQR values range from 0 to 1 and, according to the WFD, water bodies have been classified into five ecological status (ES) classes. The rating scale of EQR values was defined by [1]: 0–0.25 (bad), >0.25–0.40 (poor), > 0.40–0.60 (moderate), >0.60–0.75 (good) and >0.75–1 (high).

#### Ecological status over a decade

Longitudinal data, such as repeated measurements along time (correlation structure), are better analyzed as a linear mixed effects model allowing to include random effects and use time as a continuous variable. For this reason, we choose the linear mixed model approach to study the variability in the EQRs calculated by the CARLIT Index due to different factors, as detailed below.

A dataset reporting EQR values obtained in each WB during four different years between 2006 and 2015 was created, also including information about the operator who performed the sampling (surveyor), who could be an important source of variability, particularly in the attribution of intermediate categories (*e*.*g*. *Cystoseira amentacea* 3 and 4, see Table 1). In the mixed model, EQR values are used as a response variable and Years, WB and Surveyor as predictors. WBs, by definition, should differ in terms of their ES, but they are included as factors in the model because of their relevance as variance components. A random slope and intercept model was fitted in R with lme4 package [36] by REML. The model allows to partition variability associated to the different factors, according to the following R code:

lmer(EQR∼(1|Surveyor)+(1|WB)+(1|Year)+(0+WB|Year),data=Carlit,REML=T)

The total variance (σ^2^_T_) and variance components associated with each factor (σ^2^_x_) were estimated and then the percentage of variance explained by each factor (P_samp_) was calculated, following [37]:

$$P_{samp}=100\sigma_{X}^{2}/\sigma_{T}^{2}$$

where,

$$\sigma_{T}^{2}=\sigma_{Year}^{2}+\sigma_{WB}^{2}+\sigma_{Surveyor}^{2}+\sigma_{WBx|Year}^{2}+\sigma_{R}^{2}$$

The model was validated by plotting the residuals against the fitted EQR values. No data transformation was required. The model was chosen after validation by AIC (Akaike Information Criteria).

In order to visualize interannual changes in the dominance of the different CARLIT communities within each water body, radar charts have been produced.

#### Intra-seasonal variability

In order to evaluate variability within the spring season, EQR values from March to June 2015 were calculated in the four selected sites and, consequently, the corresponding ES values were obtained and plotted in a barplot.

## Results

### Ecological status along ten years

**Fig 2:**
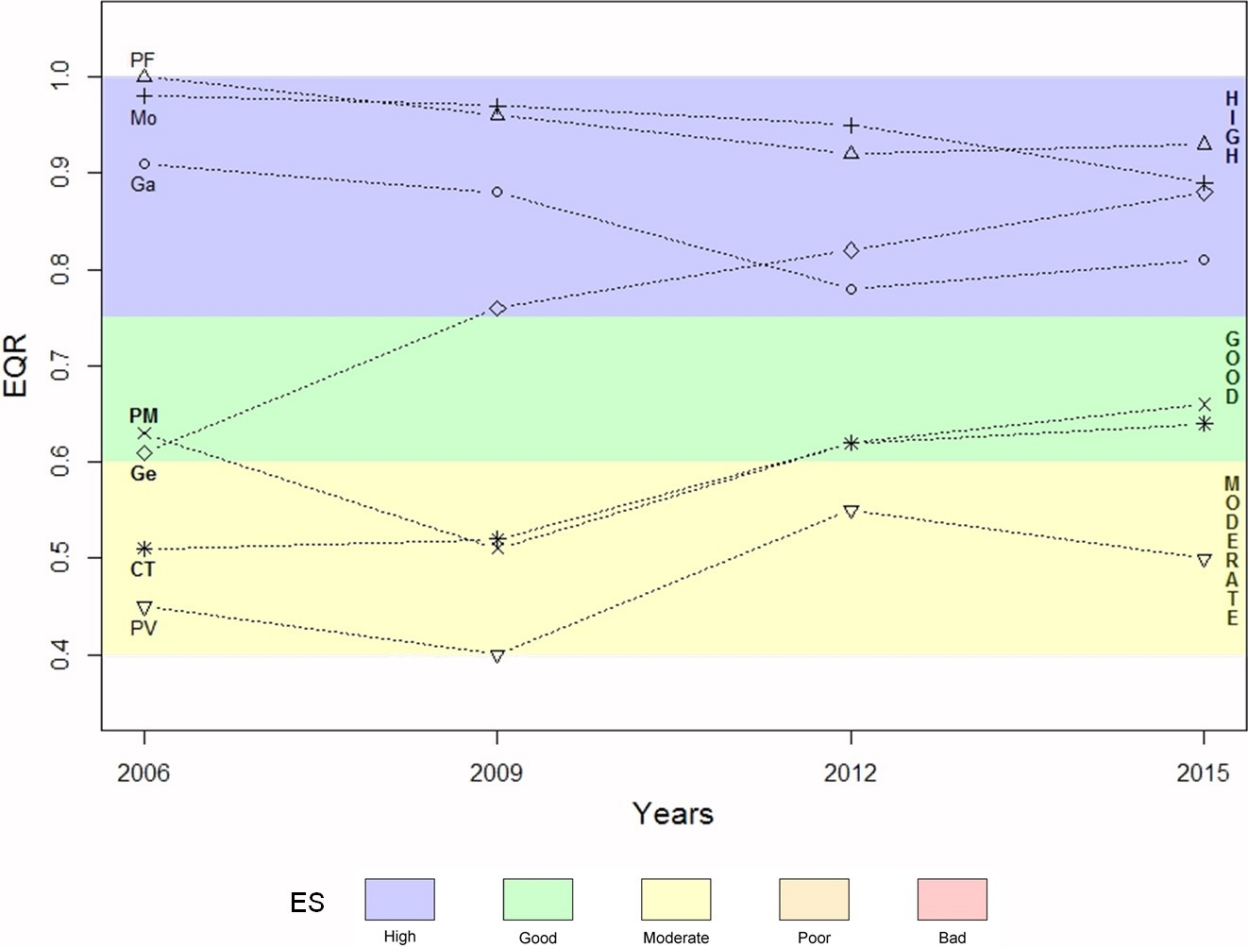
Ecological status (ES) in different years over 10 years in seven water bodies of the Ligurian coast. Data refer to CARLIT EQR values and ES for Capo Mortola (Mo), Laigueglia-Albenga (Ga), Genova-Camogli (Ge), Portofino (PF), Punta Mesco (PM), Cinque Terre (CT) and Portovenere (PV).

The ES of the Ligurian rocky shores shows overall quite positive evidences (Fig 2): 6 out of the 7 WBs are classified as “good” or “high” in 2015. In particular, over the investigated time-frame, a shift from “moderate” to “good” and from “good” to “high” has been observed for two water bodies (respectively Cinque Terre, CT, and Genova-Camogli, Ge).

The two lowest ecological status classes, “bad” and “poor”, have never been recorded along the Ligurian coast in the framework of the present study. The highest EQR scores were recorded in Portofino (PF), Capo Mortola (Mo) and Laigueglia-Albenga (Ga), slightly fluctuating along the considered years, but remaining within the “high” ecological status class. On the contrary, Portovenere (PV) displayed the lowest EQR scores and the ecological status was always maintained as “moderate” over the considered time-frame. In Punta Mesco (PM), the EQR scores fell in the “good” ecological status class, with the exception of 2009, when the ecological status decreased from “good” to “moderate”. Instead, in Cinque Terre (CT) the ecological status improved from “moderate” (2006–2009) to “good” (2012–2015). The most relevant change occurred in Genova-Camogli (Ge), where in 2006 a “good” EQR score was obtained, although close to the upper-limit of “moderate”: in the following years, since 2009, a constant increase in EQR scores was observed, showing values within the “high” class.

Linear mixed effects model results (Table 3) show that the highest percentage of variance (47%) was explained by WB, as expected (see Materials and methods section, paragraph 2.3.2). Factors Year and Surveyor, instead, did not show any associated variability (0%). Among WBs, Genova-Camogli explained the highest percentage of the inter-annual variability of the EQR values (20%). The low residual variability (7%) provides evidences that almost all variance is explained by the model.

**Table 3:** Results of linear mixed effects model fit by restricted maximum likelihood (REML). Untransformed EQR scores analyzed as a function of four random effects.

Groups	Levels	Type	Std.dev.	Variance	P_samp_^b^ (%)
**WB**^a^	7	Crossed	0.17800	0.031683	**47**
Year	4	Crossed	0.00000	0.000000	0
Surveyor	2	Crossed	0.00000	0.000000	0
Year:					
Capo Mortola			0.04112	0.001691	2
Laigueglia-Albenga			0.05730	0.003284	5
**Genova-Camogli**			0.11552	0.013276	**20**
Portofino			0.03608	0.001302	2
Punta Mesco			0.05869	0.003444	5
Cinque Terre			0.06932	0.004810	7
Portovenere			0.05869	0.003429	5
Residual			0.06637	0.004404	7

^a^WB- Water Body

^b^P_samp_- the proportion of total variance explained by each factor.

**Fig 3:**
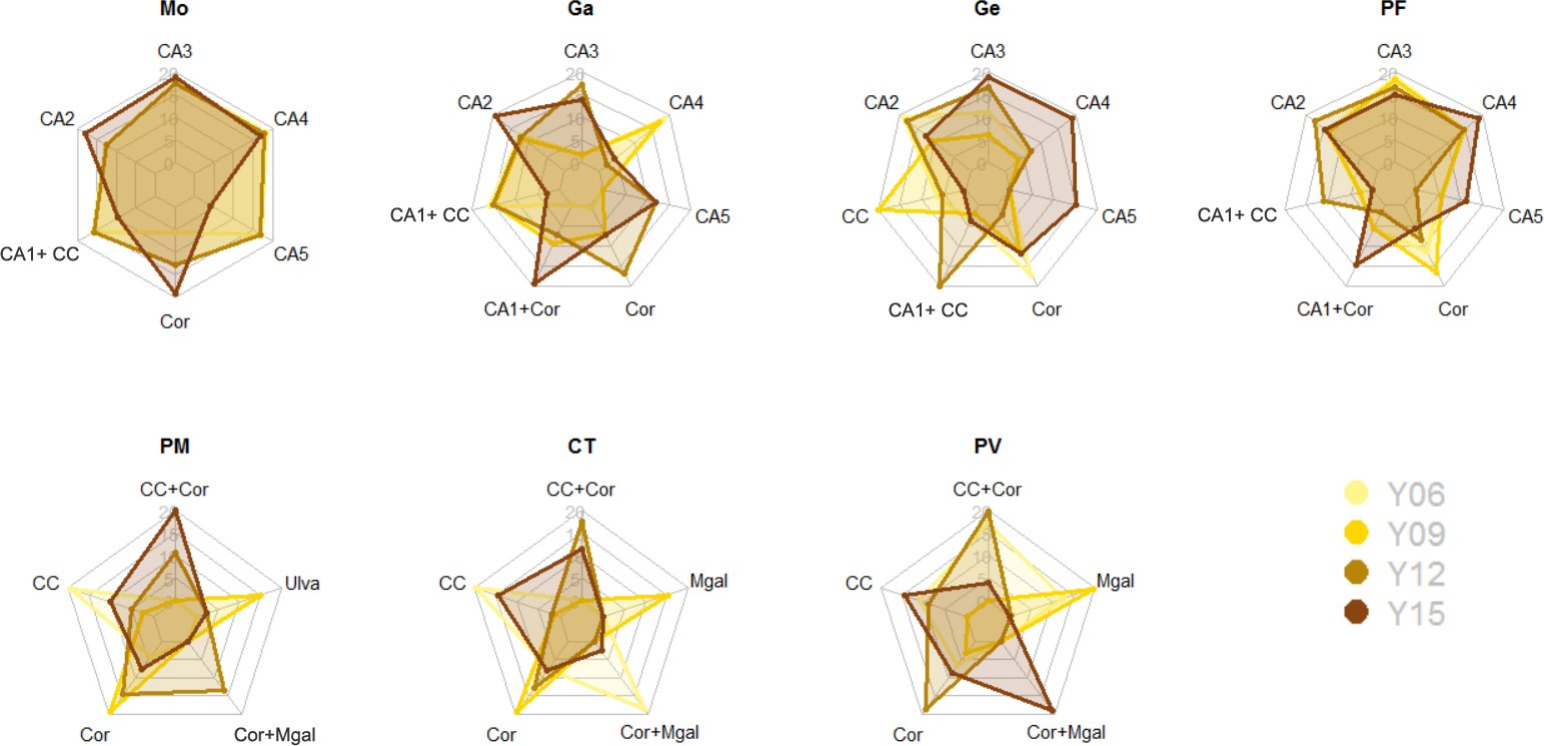
Radar charts reporting interannual changes in dominance of the different CARLIT community categories. CA1-2-3-4-5: *Cystoseira amentacea* 1-2-3-4-5; CC: *Cystoseira compressa*; Cor: *Corallina* spp.; Mgal: *Mytilus galloprovincialis*; Ulva: *Ulva* spp. in 2006 (Y06), 2009 (Y09), 2012 (Y12), 2015 (Y15) within each water body (Mo: Capo Mortola; Ga: Laigueglia-Albenga; Ge: Genova-Camogli; PF: Portofino; PM: Punta Mesco; CT: Cinque Terre; PV: Portovenere).

Interannual changes in dominance of the different CARLIT community categories (defined in Table 1) are reported in Fig 3. In Capo Mortola the coast was commonly dominated by *Cystoseira amentacea*, homogenously distributed in the different categories, from scattered plants to continuous belts (CA1- CA5). In 2015 a decrease of *C*. *amentacea* dense stands (CA3), non continuous (CA4) or continuous belts (CA5) was observed, in favor of *Corallina* spp. (Cor). In Gallinara and Portofino a similar community dominance was observed, but higher percentages of stretches of coast covered by dense stands (CA3) and continuous belt of *C*. *amentacea* (CA5) were observed in 2015 (and also 2012 for Gallinara). In the Genova-Camogli water body, in 2006 and 2009 the community was generally dominated by *Corallina* spp., *Cystoseira compressa* (CC) and scattered *C*. *amentacea* thalli (CA1-2) and progressively shifted towards stretches of coast dominated by *C*. *amentacea* dense stands or belts (CA3-4-5) in 2012 and 2015, mirrored by a substantial increase in the CARLIT Index (Fig 2).

In the easternmost side of the Ligurian coast, the only *Cystoseira* species present was *C*. *compressa*, dominant along most of the coast in Punta Mesco and Cinque Terre in 2006. This community (CC) shifted to a mixed one with *Corallina* spp. in the last years (CC+Cor). A concurrent decrease of *Ulva* sp. dominance in Punta Mesco and of *M*. *galloprovincialis* (Mgal)in Cinque Terre was observed. In Portovenere a constant massive presence of *M*. *galloprovincialis* was observed, with a relevant decrease in 2012 and a shift to a co-dominated community with *Corallina* spp. (Cor+Mgal) in 2015.

Scores of the anthropogenic pressures on the coast, LUSI and MA-LUSI-WB indices are reported in Table 4, alongside EQR and ES values according to CARLIT in 2015. All the pressure indices are relatively low, as well as LUSI and MA-LUSI-WB, providing evidence of a good agreement with the EQR values. Yet, given the low number of considered WBs, it was not possible to test any significant statistics.

**Table 4:** Scores of the anthropogenic pressures according to LUSI and MA-LUSI-WB indices, the EQR and ES values obtained in 2015 by CARLIT Index.

WB^a^	Urb^b^	Ind^c^	Agr^d^	FW^e^	Coast^f^	LUSI	MA-LUSI-WB	EQR	ES
Mo	1	0	2	0	0.75	2.25	5.98	0.89	High
Ga	1	0	0	0	0.75	0.75	4.92	0.81	High
Ge	3	0	1	0	1	4	8.11	0.88	High
PF	1	0	0	0	0.75	0.75	3.39	0.93	High
PM	1	0	0	0	0.75	0.75	3.92	0.66	Good
CT	1	0	2	0	1	3	5.92	0.64	Good
PV	1	0	3	0	1	4	8.13	0.50	Moderate

^a^WB- Water body

^b^Urb- Urban area

^c^Ind- Industrial area

^d^Agr- Agricultural area

^e^FW- Freshwater

^f^Coast- Type of coast (concave, convex or straight).

### Intra-seasonal variability

**Fig 4:**
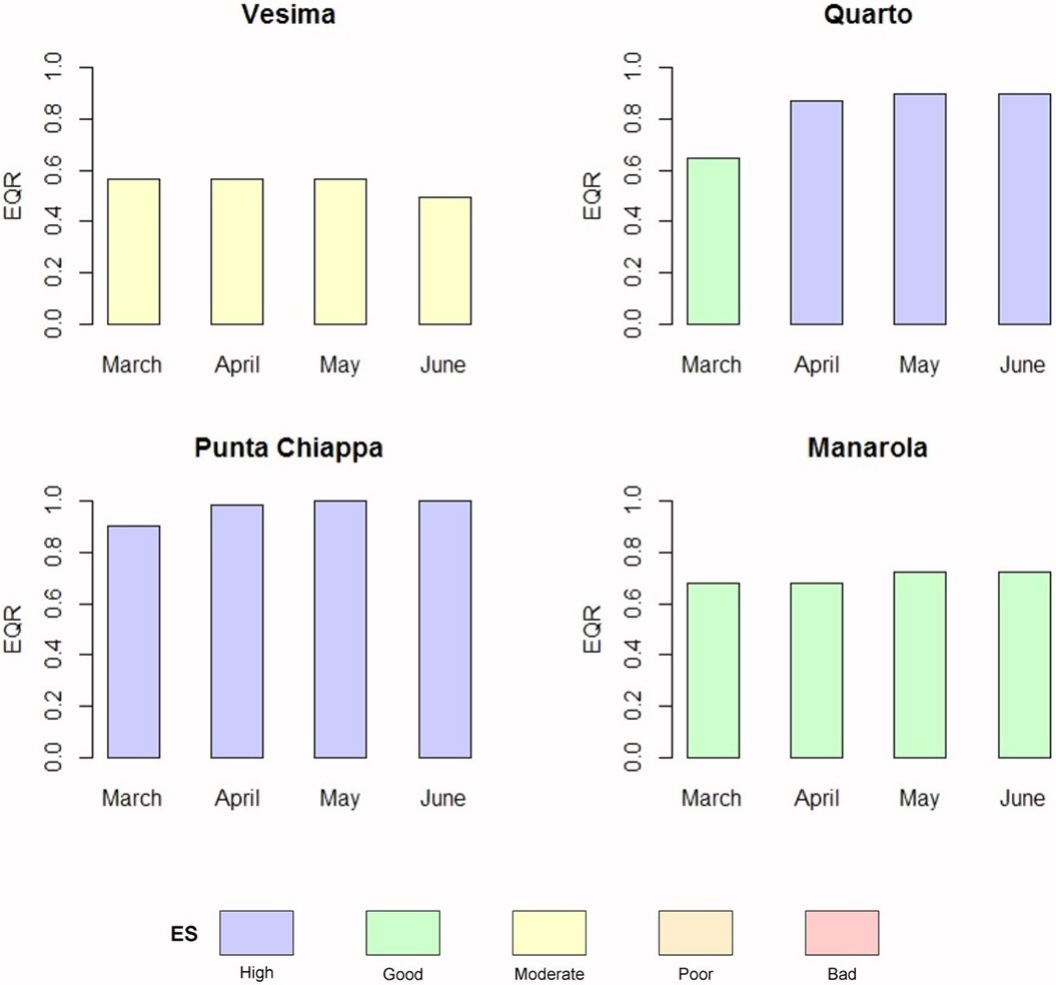
Ecological status of the four selected sites along the Ligurian coast, for each month during the spring season 2015, for the intra-seasonal variability assessment.

The EQR scores obtained in each site from March to June, shown in the bar plots (Fig 4), fell in the same ecological status class in the different months within each site, with the exception of Quarto dei Mille. In this site, the values showed a different ecological status in March (“good”) compared to April, May and June (“high”).

## Discussion

As required by the Water Framework Directive (WFD, 2000/60/EC), all the Ligurian water bodies considered in the present study (with only one exception) reached the “good” ES by 2015. In addition to the achievement of the WFD’s goal, an encouraging general improvement of the ES along the Ligurian coastline has been observed in the considered decade (Fig 2 and Fig 3).

A general improvement of the ecological quality, based on CARLIT index, can be also observed at a larger scale in the North-Western Mediterranean basin according to a recent study by [4], that additionally underlines the importance of considering a combination of different indices, as required by the WFD, in order to account for the ecological quality of a water body as a whole, the status of the ecosystems and natural and human-induced pressures.

The general increase in ES observed along time in the study area is actually mirrored by an improvement of coastal water quality, as proved also by other indices based on different key biological elements (*e*.*g*. AMBI Index on macroinvertebrates, S1 Fig) and bathing water quality (*i*.*e*. quantification of *Enterococcus* spp. and *Escherichia coli*, S2 and S3 Figs).

During the first CARLIT assessment performed along the Ligurian coast, scores below the “good-moderate” threshold were recorded in the Genoa urban area and in the sites at the easternmost side of the region [2,6], probably because of the low abundances or lack of the most sensitive macroalga considered by the Index, *Cystoseira amentacea* (Table 1).

In the present study we highlighted a particularly remarkable trend in Genova-Camogli WB (Ge), where the ES recorded in 2006 was close to the lowermost limit of the “good” class (EQR = 0.61), while in 2015 it was classified in the uppermost boundary of the “high” class (EQR = 0.88). Since no relevant artificial modification in coastal morphology (*e*.*g*. ports, breakwater, etc) and human pressure occurred in the area (both reported by [34] as possible sources of change in ES), the improvement in ES should be ascribed to an actual amelioration in water quality, mirrored by an increase of the abundance of macroalgae sensitive species. At the start of the study, the communities were generally dominated by intermediate sensitive species (*i*.*e*. *Corallina* spp., *Cystoseira compressa*) and scattered *C*. *amentacea* thalli, progressively shifting during the considered decade towards dense stands or belts of *C*. *amentacea* (Fig 3). This amelioration could be related to the water treatment enhancement occurred in the last years (*i*.*e*. intervention on drainage pipe following two relevant flood events occurred in the area in 2011 and 2014 and improvement of sewage treatment plant in the last years performed by the competent company IRETI S.p.A.). The CARLIT index resulted effective in detecting changes in ES possibly related to local anthropogenic pressures in a relatively short time scale, where anthropization indices (*e*.*g*. LUSI, MA-LUSI-WB) would have failed, given the lack of significant changes in the considered pressures (*e*.*g*. land use, freshwater) along a decade in the use of the Ligurian coastline.

*C*. *amentacea* is still completely missing in the easternmost side of the Ligurian coastline starting from Punta Manara, around 20 km North-West from Cinque Terre (authors personal observation). This species has been recorded until the end of the 19^th^ century (authors personal observation through herbaria records, S4 Fig) and its disappearance in the area can be explained by the habitat fragmentation due to coastal developments and water pollution and high sediment loads caused by intense excavating activities for extracting construction material (mostly for building railway and highway in the area), that occurred in the first half of the 20^th^ century. Even if such disturbing activities have largely been reduced in the last decades, with significant changes in the riverine basin and sediment load to the sea, and a Marine Protected Area has been established (1997), this sensitive species is still completely absent along these stretches of coast. This is probably due to its low dispersal capacity (< 1m [38–41]) that hampers the natural recovery of this species, additionally affected by other local factors such as mussel farming (competition for space with the settled mussel juveniles).

Despite the absence of the species with the highest sensitivity level (*C*. *amentacea*), ES values belonging to the “good” class have been recorded also in this area, because of the increased percentage in the last years of stretches of coast dominated by species with intermediate sensitivity levels (*i*.*e*. *C*. *compressa*, and *Corallina* spp.), alongside with a decreasing trend of less sensitive species (*Ulva* spp. in Punta Mesco and *Mytilus galloprovincialis* in Cinque Terre), with the exception of Portovenere which still remains in the “moderate” ES class, not fulfilling WFD’s requirements. Low ES values may be ascribed to the proximity to large mussel farms and present activities carried out in the Port of La Spezia, such as shipbuilding, shipping, yachting and tourism, whose wastes are accumulated and driven along shore by the superficial current of the Gulf of La Spezia and the main current of the Ligurian Sea [42]. A similar effect of the port area and current regime on ES values is observed on the Western side of Genoa Port (Vesima, “moderate” class Fig 4).

Several factors are considered as sources of error in the different quality indices, which use marine macrophytes as Biological Quality Elements [4,6,34,43–45]: spatial variability (*e*.*g*. sites, depth), temporal variability, inter-annual variability and human associated error.

The spatial variability, due to horizontal and depth-related heterogeneity, may be erroneously incremented by limited sampling designs not representative of entire WBs. In several cases, such variability has been identified as one of the most important causes of misclassification of ES. This possible confounding element leads to the strong recommendation to carry out monitoring at large spatial scales [45]. Specifically, the CARLIT Index in the Ligurian Sea has been implemented following a hierarchical design, encompassing three different and representative stretches of coast (more than 1000 m long) in each water body, as performed in the ARPAL implementation of the index, following ISPRA methodological report indications [28]. This suggests that the among water bodies variability observed and reported in the present study is reliable, unaffected by spatial heterogeneity and ascribable to actual ES differences.

The temporal variability of EQR values, in terms of natural inter-annual variability, has been addressed in several previous studies using different indices, which reported that it may be considered very low, ranging between 0% and 15% [44]. Specifically, for the CARLIT Index, two studies already supported low inter-annual variability. Asnaghi et al. [6] did not find inter-annual differences comparing ES measured along the Ligurian rocky coast in two consecutive years, and [4] obtained similar results along French coasts, performing an assessment in two different periods (encompassing three years each) on a longer time scale. Also in the present study no inter-annual significant differences have been observed (0% of variability associated to the factor “Year”; Table 3). The outcomes of these studies support the effectiveness of performing CARLIT monitoring at a lower temporal resolution, *e*.*g*. once every three years for the whole water body (as currently implemented by the Ligurian Environmental Agency). On a longer time-frame, data collected every three years, instead, show trends that may be related to actual changes in ES of individual water bodies (Figs 2 and 3).

In the present study, temporal variability has been addressed also in terms of intra-seasonal variability. Although the recommended period to perform the CARLIT Index assessment in the NW Mediterranean is spring (April–June [1]), peculiar seasonal environmental conditions that trigger macroalgal development, such as light intensity and temperature, are not stable within the season time-frame and consistent across years. This intrinsic variability of environmental conditions may affect the growth of macroalgal species, potentially influencing the ES attribution. Additionally, unfavourable meteo-marine conditions may force to delay samplings along the season, although within the defined spring time-frame. Over such time-frames, the abundances and the morphology of the macroalgal species of the rocky shores, particularly for the canopy forming *Cystoseira* spp., could be largely variable. Sampling before or after the maximum macroalgae growth peak, may possibly cause a misclassification of the community category, affecting the EQR calculation.

In the present study, no variability in the classification into ES classes during the whole April-June time-frame emerged for all the investigated water bodies, supporting the appropriateness of the whole recommended time-frame for the implementation of the CARLIT Index (Fig 4), since intra-seasonal variability does not hamper the attribution of a consistent community category.

In a long term comparison perspective, the possible bias related to the operator (surveyor) subjectivity is an additional factor to be taken into account in assessing community category. Such effect was tested in the present study through the REML model, for the first time for the CARLIT Index, showing that operator-associated variability did not affect the uncertainty of the EQR calculation (0% of variability associated to the factor “Surveyor”; Table 3) and consequently the classification of water bodies into the different ecological status classes along years. The same result has been obtained also for other macrophyte-based quality indices [43,44].

## Conclusions

Though the present study, the reliability of the observed general improvement of the ES over a decade along the Ligurian coasts has been addressed, teasing apart potential sources of variability associated to CARLIT index implementation.

The reliability of quality indices is essential to comply with the requirements of marine environment directives, in order to correctly classify water bodies ES with an appropriate level of confidence. Hence, teasing apart any natural annual variability, spatial variability or human related one due to the operator is fundamental in order to establish a good long-term monitoring programme to be carried out by environmental regional agencies.

The present study elucidates different aspects linked to the variability of the CARLIT Index, in order to validate procedures carried out by monitoring agencies, and provides an overview of the ecological status of the Ligurian coasts. Our main findings are: i) an increase of *Cystoseira* spp. detected along the Ligurian coast in the last decade, in particular along Genova-Camogli stretch of coast, probably linked to a higher attention in waste-water treatment; ii) an improvement of the ES of almost all of the WB studied (six out of seven) in the Ligurian coast; iii) only one WB, Portovenere (PV), did not yet fulfill the WFD requirements for 2015, being classified as “moderate”, possibly as a consequence of present and past heavy anthropogenic impacts and limited dispersal of the most sensitive species, preventing self-recruitment; iv) the lack of intra-seasonal and operator effects in the estimation of the ES, supporting the reliability of the CARLIT Index implementation carried out by the Ligurian Environmental Agency.

These results support the reliability of monitoring procedures carried out though the CARLIT Index and highlight the need and the effectiveness of reduction measures for anthropogenic impacts in order to achieve the ES required by European directives. The understanding of mechanisms that affect *Cystoseira* spp. distribution is a relevant issue in the light of monitoring and assessing potential changes in the Ecological Status assessment of Mediterranean rocky shores. Moreover, the implementation of CARLIT will provide a benchmark for the assessment of *Cystoseira* species distribution and abundance. Its application along years will build a long-term dataset collected using a standardized methodology, which will be useful to assess the progression or regression of *Cystoseira* species with the aim of conservation [11], management and eventually restoration.

## Supporting information

S1 Fig AMBI index values for the investigated Sites in 2009 and 2015 (data available on ARPAL website: www.ambienteinliguria.it). (TIF)

S2 Fig Abundances of *Enterococcus* spp. (mean value + standard error) for the investigated Sites in 2009 and 2015 (data available on ARPAL website: www.ambienteinliguria.it). (TIF)

S3 Fig Abundances of *Escherichia coli* (mean value + standard error) for the investigated Sites in 2009 and 2015 (data available on ARPAL website: www.ambienteinliguria.it). (TIF)

S4 Fig Occurrence of *Cystoseira amentacea* along the Ligurian coast from herbaria records at the end of 19^th^ century (A) and nowadays (B).
(TIF)
