# Supplementary figures and images for: Ecological status improvement over a decade along the Ligurian coast according to a macroalgae based index (CARLIT)
Source: PLoS One. 2018 Dec 31;13(12):e0206826. doi: 10.1371/journal.pone.0206826 (PMC6312225; doi:10.1371/journal.pone.0206826)

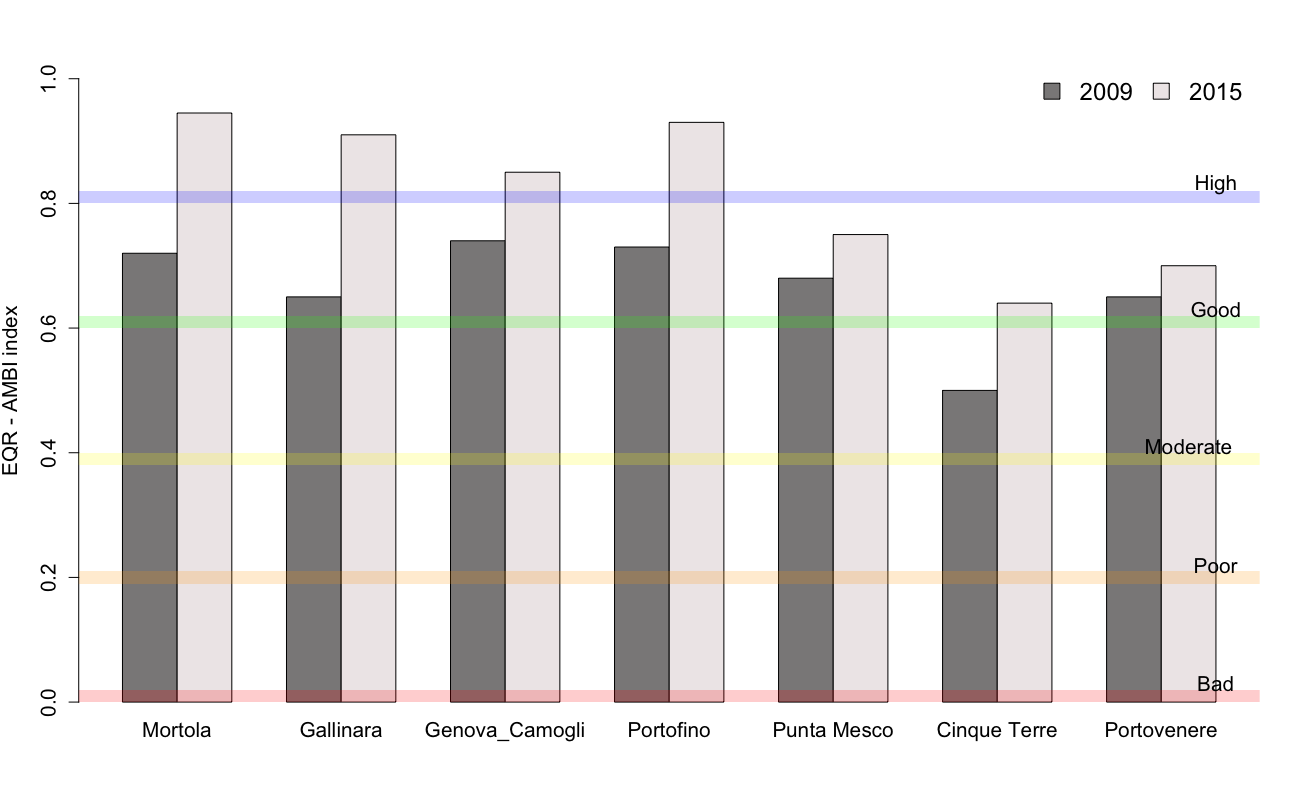

Supplement: S1 Fig — (TIF) [file pone.0206826.s001.tif]

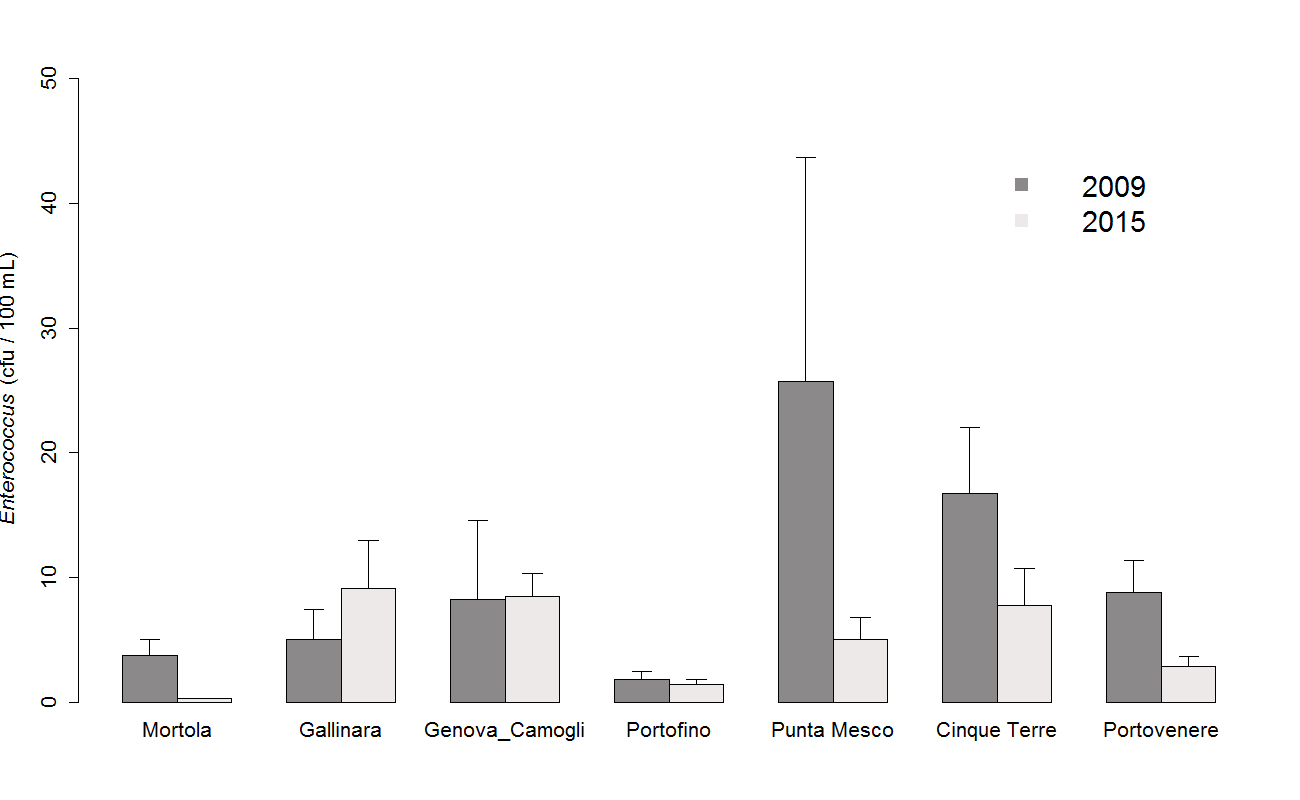

Supplement: S2 Fig — (TIF) [file pone.0206826.s002.tif]

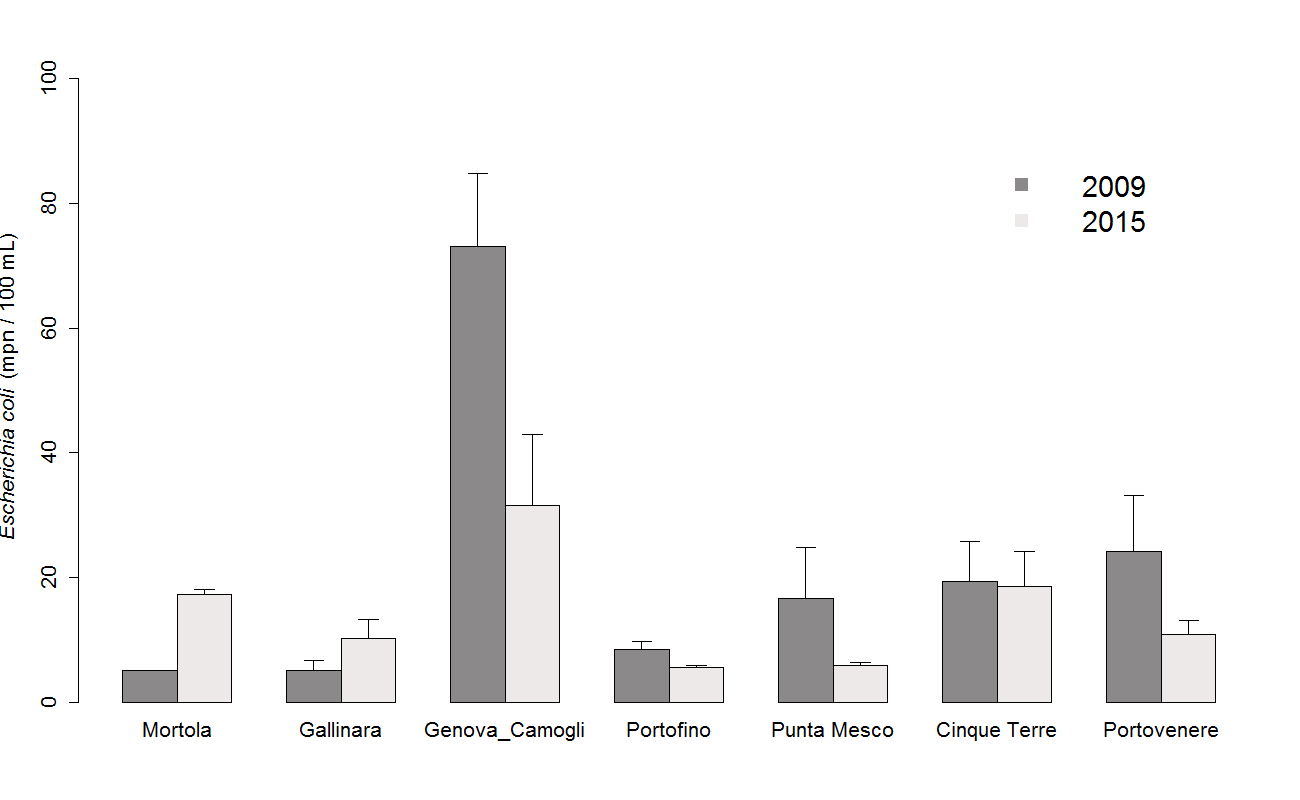

Supplement: S3 Fig — (TIF) [file pone.0206826.s003.tif]

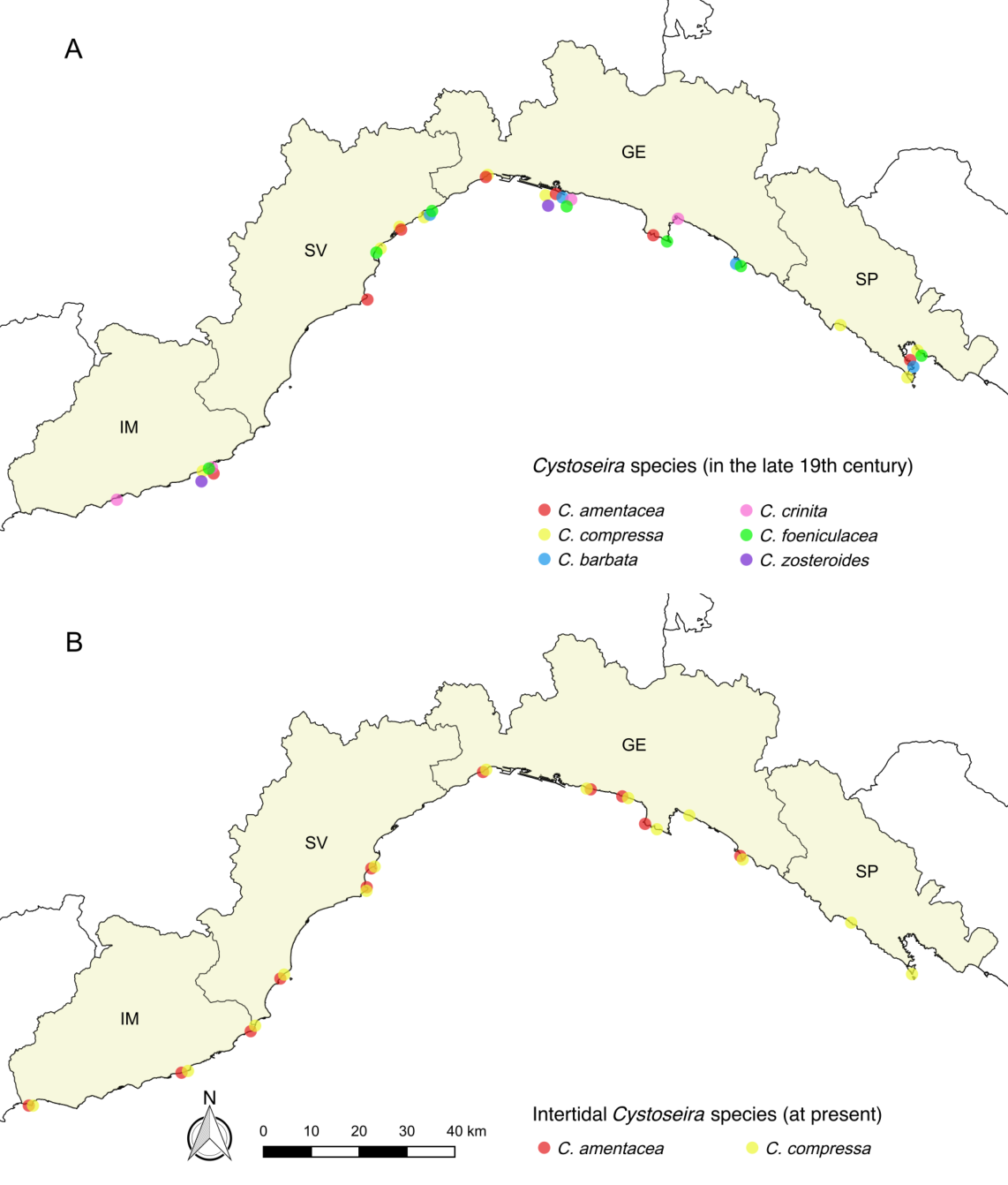

Supplement: S4 Fig — Occurrence of Cystoseira amentacea along the Ligurian coast from herbaria records at the end of 19th century (A) and nowadays (B). (TIF) [file pone.0206826.s004.tif]
